# Transactions of the Society for the Encouragement of Arts, Manufactures and Commerce, for the Session 1833-1834; Being Part I. of Volume L

**Published:** 1835-04-01

**Authors:** 


*Transactions of the Society for the Encouragement of Arts, Manufactures, and Commerce, for the Session* 1833-1834; *being Part* I. *of Volume* L.—London, 1834. 8vo. pp. 238.

This is an interesting volume, but the major part of it is, of course, alien to our Journal. Here and there, however, points are treated of, not unsuited to a Medical Review. We will cull a few of these for the gratification of our readers.

Mr. Webster has communicated an account of the effect produced on potatoes by immersion in ammoniacal water, or in brine. The potatoes are to be immersed for four or five days in a solution containing an ounce of liquor ammoniæ to a pint of water: this kills the germ, without spoiling the tuber as an article of food. This is a great advantage, when it is desired to export them to tropical climates, or use them in long voyages. If the immersion is extended to three weeks,

"The potato becomes tough and shrivelled while in the liquor, and, when dried by exposure to the air, assumes quite a new form, as evidenced by the specimens transmitted. The potato appears consolidated, and the qualities of the potato are greatly lost; for, on boiling, it assumes the appearance of sago or starch, yet still firm, and retaining its form. But, if used in its dry and uncooked state, it has a mealy flavour, and the properties of corn.

"There is no chemical change effected on the potato, but merely a mechanical consolidation and extraction of moisture; for precisely the same effect may be produced by immersing potatoes in strong solution of salt and water, taking great care to remove, by subsequent ablution, the whole of the salt; and this requires some time, and repeated changes of water. There is a small packet of potatoes consolidated by salt and water, and some of the meal produced therefrom. This mode may perhaps be found applicable to the conversion of refuse potatoes into food for cattle or pigs, being sweet, good, and wholesome, and very nutritious, dry diet. The mode is cheap, and within the reach of every one.

"The hydrate of potato-starch forms a very tolerable tapioca for puddings, of which I forward a specimen." (P. 23.)

Mr. T. N. R. Morson, on examining some English opium rewarded by the Society, found it "very inferior in strength to Turkey opium, and not unlike some of the bad varieties of Egyptian: it contains codeine, and a larger proportion than usual of meconic acid. Twenty ounces avoirdupois of the dry opium contain

384 grs. of pure morphia,
220 grs. of narcotine." (P. 25.)

Captain Bagnold, R.N., has invented an anatomical puncturing forceps, for preventing accidents in sewing up bodies after post-mortem examinations. He says,

"The forceps are to be held in the left hand; the lower or projecting jaw pushes back the fat under the skin, and, when the instrument is closed, a puncture is made through the integuments. A slight pull by the hand of the operator enlarges the puncture sufficiently to allow the passage of a blunt needle, instead of a pointed one. The instrument is suffered to expand by means of its spring; and, a hold being again taken on the opposite flap of the incision, the ligature is again passed; and the operator may place the stitches within one eighth of an inch of each other, if he thinks it requisite, without the necessity of once bringing his fingers in contact with the subject, and in half the time that it can be done by the needle as hitherto used. A veterinary surgeon, to whom I have shown it, is of opinion that it is admirably adapted for sewing up incised wounds in horses and other cattle, whose strength and restlessness renders the operation difficult with the usual needle." (P. 65.)

We have not seen these forceps, but the recommendations of Mr. Lane, the well-known anatomist, and Mr. Hutchins, the able apothecary of St. George's Hospital, which are appended, will be certain to secure it a favourable examination from the profession.

Mr. Forster has made an improvement in trusses, which is stated to consist in "the insertion of a spiral spring, acting by compression, at the base of the strap, whereby the instrument adapts itself to the varying diameter of the part round which it is applied, and thus preserves an equal pressure on the pad. It has been applied with signal advantage to persons in the habit of raising great weights, such as millers' men, to whom the common truss soon ceases to afford any support." (P. 68.)

